# The bikini incision anterior cemented total hip arthroplasty: Assessment of radiological and clinical outcomes: A mid-term review

**DOI:** 10.1051/sicotj/2020050

**Published:** 2021-01-12

**Authors:** Ikram Nizam, Avinash Alva, Sophia Gogos

**Affiliations:** 1 Ozorthopaedics – Centre for Adult Joint Arthroplasty 1356 High Street Malvern VIC 3144 Australia; 2 AOA Accredited Fellow-Hip, Knee and Sports Surgery, Mulgrave Private Hospital Blanton Drive Melbourne VIC 3170 Australia; 3 Monash University Surgical Interest Group Scenic Blvd & Wellington Road Clayton VIC 3800 Australia

**Keywords:** Bikini incision, Bikini hip replacement, Direct anterior approach, Hip arthroplasty, Femoral cementation

## Abstract

*Introduction*: There has been an increased interest in minimally invasive direct anterior approach total hip arthroplasty (THA) to provide greater patient satisfaction, improve pain relief, and reduce the duration of hospitalisation. A direct anterior approach hybrid cemented THA, utilising a bikini line incision, can be technically challenging. We aimed to undertake radiological analysis of femoral stem cementation, clinical outcomes, and component survivorship. *Methods*: Over a 5-year period, 215 primary elective bikini anterior THA conducted by a single surgeon were included. All procedures were performed using a cemented collarless polished stem. The operation was performed on a standard operating table. Patients undergoing posterior approach, revision procedures, and fractured neck of femurs were excluded. Post-operative radiographs were analysed for femoral cementation quality using the Barrack grading system. Harris hip scores (HHS) were determined at 6 weeks, 12 weeks, annually thereafter and the difference in HHS was noted. *Results*: In total, 215 anterior bikini THA (*R* = 101, *L* = 114) were performed in 199 patients (*M* = 89, *F* = 110) with a mean age of 77 and mean follow up of 2.9 years (range = 0.5–5). Radiographic analysis of femoral cementation showed 189 femoral stems (88%) were either Barrack A or B cementation grade, suggesting optimal cementation. Lucency in the cement-bone interface occurred mainly in Gruen Zone 1 (43%) and Zone 13(46.9%). At the most recent follow-up (mean 2.9 years), component survivorship was at 99.54% (stem). Significant improvement was noted in Harris hip scores at final follow-up (from 54 preoperatively to 92.7 at 2.9 years postoperatively). *Conclusion*: Our results suggest that a bikini incision direct anterior approach for total hip arthroplasty can be safely employed to perform cemented femoral stems on a standard operating table.

## Introduction

As the incidence of primary and revision total hip arthroplasty (THA) rises, there is renewed clinical interest in factors that may contribute to patient satisfaction including the length of hospital admission, pain control, early resumption of pre-morbid activities, and surgical revision [1–3].

Recent studies have suggested that direct anterior THA results in better pain control, shorter hospital stay, improved gait speed, hip flexion at 3 months, early return to driving, reduced dislocation rate, and earlier discontinuation of assisted ambulatory devices compared to posterior THA [1–6]. The bikini incision for DAA follows the anatomic skin crease resulting in improved healing, shorter and narrower scars with better aesthetic appearance [5, 7]. However, DAA THA has been associated with complications such as temporary and chronic upper thigh dysaesthesia, complications in patients with high BMI or complex anatomy, trochanteric, and calcar fractures [7–9].

Cementation of femoral components in total hip replacements has shown excellent survivorship at 20 years follow-up, with low rates of revision [10–12]. Stem design, cement type, cementation technique, patient age, and underlying disease have been reported to affect outcomes of cemented THA [13, 14]. During the anterior approach, the trajectory of the femoral canal may result in the prosthesis passing close to the lateral cortex [15]. Although cadaveric studies have demonstrated no compromise in the cement mantle during DAA THA, there is currently a lack of clinical data regarding femoral cementation in bikini incision anterior THA and component survivorship [15]. The primary aim of this study was to determine if femoral cementation and component positioning were satisfactory using a bikini anterior approach THA on a standard operating table. The secondary aims were to measure clinical outcomes and component survivorship at the most recent follow-up.

## Methods

**Figure 1:**
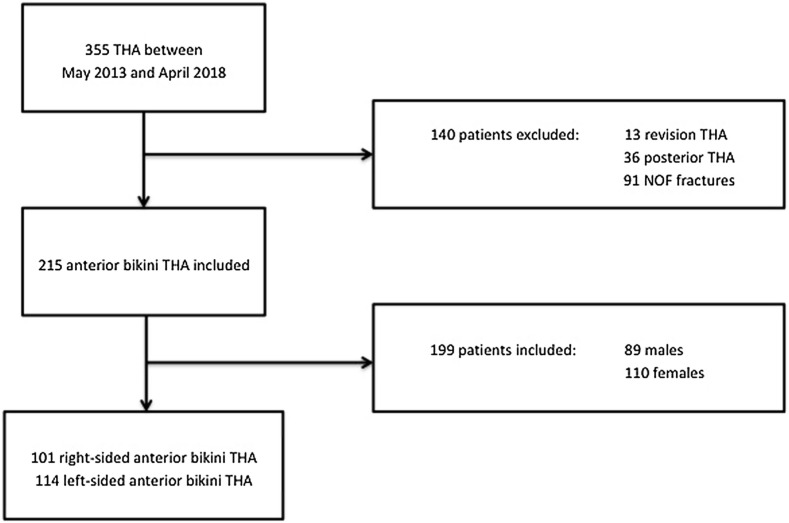
Flowchart.

All consecutive patients who underwent elective bikini anterior THA by a single surgeon in one institution between May 2013 and April 2018 were included (Figure 1). Indications for surgery included osteoarthritis, avascular necrosis, posttraumatic arthritis, and post-Perthes sequelae. Informed consent was obtained from patients and the study was approved by the local institutional review board. Patients undergoing posterior approach THA and revision THA were excluded. The posterior approach was utilised for fracture neck of femur, compromised skin quality in the lateral groin crease, presence of stoma bags, concomitant gluteus tendon tears requiring repair, and complex congenital/post-traumatic dysplasia. All patients undergoing the bikini THA were treated with the same operative technique, perioperative care, and post-operative rehabilitation protocol with early mobilisation, as previously described in the literature [7, 16].

Patient demographics including age, sex, BMI, operative side, preoperative Harris hip scores (HHS) and indication for surgery were obtained from the institute database as recorded at the time of surgery or preoperatively. All patients were reviewed at 2 weeks, 12 weeks and annually post-operatively. HHS was recorded during the follow-up period.

A retrospective radiological analysis was conducted using post-operative X-rays conducted at the most recent follow-up. Anteroposterior (AP) views of pelvis X-ray and lateral view of the hip were used in this review. Stem positioning and radiological lucency at the cement-bone interface were determined using the Barrack grading system and Gruen zones [17, 18]. Measurements were also taken of the angle between the long axis of the femoral stem and the anatomical axis of the femur to assess stem position. Leg length discrepancy was measured on AP Pelvis X-ray as the distance from the line at the inferior aspect of the ischial tuberosities to the most prominent medial point of the lesser trochanter [19]. All measurements were conducted by an experienced orthopaedic fellow. The inteleviewer PACS system (Intelerad Medical Systems Incorporated, Quebec, Canada) was used to review X-rays and measure stem positions.

## Surgical procedure

All patients underwent a vessel-sparing bikini anterior THA on a standard operating table as previously described [7, 16]. Cemented femoral components (CPCS Smith and Nephew, Memphis TN) were used if *T*-score was less than or equal to −2.5, Dorr type C femurs, currently taking steroids or anticoagulant, in patients where poor bone quality was suspected by the operating surgeon during femoral broaching and preferred in patients who were over 75 years old. Fourth-generation cementation technique was utilized (medullary plug, pulsatile lavage, vacuum mixing, cement gun, distal centralizer, and a proximal rubber seal to pressurize cement). Simplex HV (high viscosity) with Gentamycin cement was used (Stryker Howmedica Osteonics, USA). We used a flexible disposable introducer to place the cement restrictor. In addition, we utilized a cement gun with flexible nozzles in all our cases, which enabled easy cementation followed by pressurization before insertion of the definitive stem (Smith and Nephew, Memphis, TN, USA).

Leg lengths at the medial malleoli were measured with a squared pelvis during trialling, taking into account any fixed flexion deformities in the contralateral hip or knees. Oxinium femoral heads (Smith and Nephew Memphis, TN), R3 three-hole hydroxyapatite (HA) coated Acetabular shell and XLPE lipped Liner. (Smith and Nephew Memphis, TN) were used in all cases. Local infiltrative anaesthesia using an intraarticular catheter for 24 h postoperatively routinely used as previously described by Kerr and Kohan [20]. Skin closure was achieved using Monocryl monofilament absorbable sutures and a thin Comfeel dressing applied.

## Results

Over the 5-year study period 215 anterior bikini THA (101 = right, 114 = left) were performed in 199 patients (*M* = 89, *F* = 110) with a mean age of 77 (range = 75–92 years), average BMI of 28.6 (range = 23.2–34.9) and follow up of 2.9 years (range = 0.5–5.9). Overall 12 patients had a follow up period of less than one year. The average preoperative Harris hip score was 54 (range = 44–59), increasing to 92.7 (range = 64–100) at final follow-up.

**Figure 2:**
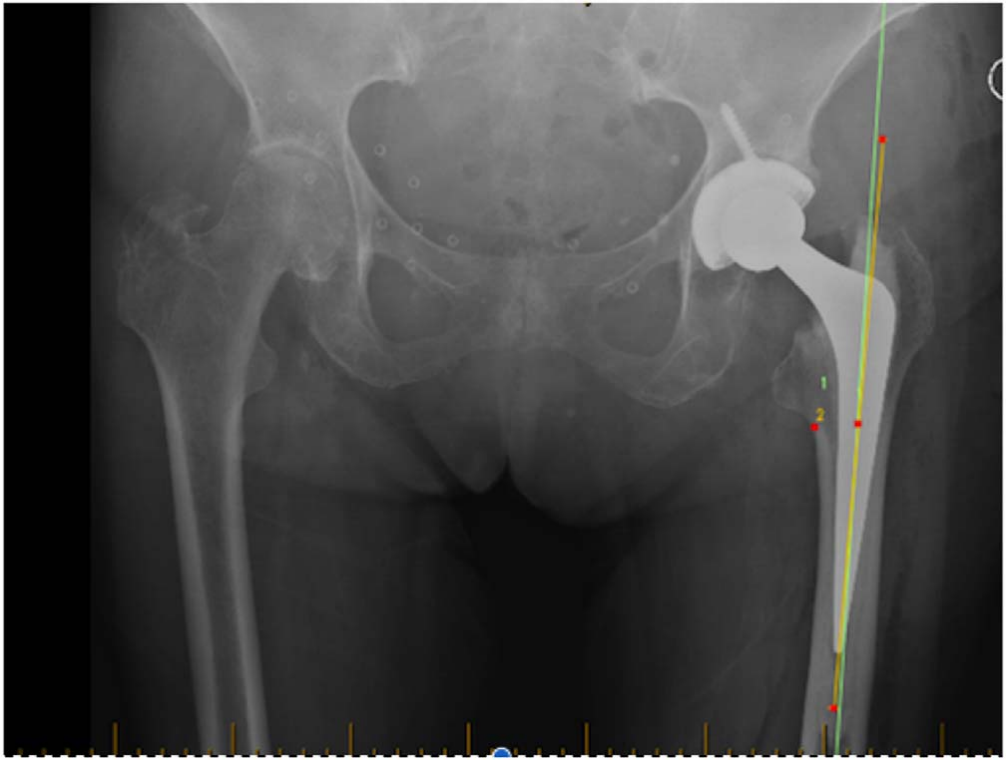
Anteroposterior view of X-ray hip showing a Barrack grade A cementation following bikini total hip replacement

Radiographic analysis of femoral cementation showed 47 femoral stems (21.95%) were Barrack A (Figure 2), 142 femoral stems (65.85%) were Barrack B, and the remaining 26 stems were recorded as Barrack C (Table 1). Lucency in the cement-bone interface occurred most commonly in Gruen Zone 1 (43%) (Table 2). The average angle between the femoral stem and anatomic axis of the femur was 0.41° of varus (range = 2.77° valgus to 6.39° varus).

**Table 1:** Results of Barracks grading of cementation (absolute values and percentage).

Barrack grading	Number of hips (%)
A	47 (21.95)
B	142 (65.85)
C	26 (12.19)

**Table 2:** Radioluscent lines on X-rays in each Gruen Zone (AP and lateral views; absolute values and percentage).

Gruen zone	Number of cases with lucency	Radiolucency lines (%)
Zone 1	92	43
Zone 2	40	19
Zone 3	36	17
Zone 4	30	14
Zone 5	21	9.7
Zone 6	1	2.4
Zone 7	47	21.9
Zone 8	45	20.9
Zone 9	35	16.2
Zone 10	55	25.5
Zone 11	9	4.1
Zone 12	83	38.6
Zone 13	101	46.9
Zone 14	45	20.9

There were no dislocations or incidences of deep venous thrombosis or pulmonary emboli. Ten (4.6%) patients reported temporary thigh paraesthesia of varying distribution due to neuropraxia of the lateral femoral cutaneous nerve (LFCN), however, all had resolved by 18 months post-operative.

There were two cases (1.0%) of thigh hematoma, which resolved spontaneously, with one patient inadvertently started on both enoxaparin and high dose aspirin. Two patients (1.0%) had leg length discrepancies of less than 11 mm noted at the time of surgery of which one patient had a shortened contralateral leg as a result of posterior THR done at a different centre. One patient (0.5%) had a traumatic Vancouver type B2 periprosthetic fracture 19 days post-operatively after falling down a flight of stairs while walking unaided 18 days after surgery. Overall stem survivorship at 12-month follow-up was 99.54% and cup survival was 100%. One cemented stem was revised at three weeks post-surgery after a traumatic fall and one case was lost to follow-up at 18 months post-surgery.

## Discussion

Our study represents the largest radiographic analysis of femoral cementation in bikini anterior THA by a single surgeon on a standard operating table. The radiographic analysis demonstrated satisfactory cementation and component positioning at medium-term follow-up, with 47 (21.95%) Barrack A femoral stems and 142 (65.85%) Barrack B femoral stems.

Barnett et al. reported a 0.84% incidence of fractures in over 5000 anterior approach hip arthroplasties [9]. Overall, 95% of their cases utilised uncemented femoral stems. Although an anterior approach THA is suggested to be associated with increased postoperative complications, some current literature does not demonstrate increased overall complication rates compared to other surgical approaches [7]. Nevertheless, there are studies demonstrating high failure rates of the femoral components potentially due to the technically challenging femoral exposure [21].

Due to the technical difficulty associated with femoral preparation during the anterior approach, it is believed that cementation quality may be compromised. Adequate femoral exposure is critical to perform satisfactory cementation. However, the exposure may be compromised due to incomplete soft tissue releases, poor limb positioning, or unsatisfactory placement of retractors. Poor exposure and inadequate releases result in inadequate femoral elevation leading to abnormal trajectory when inserting the stem after cement pressurization. Leunig et al. reported satisfactory component positioning in bikini anterior THA with cementation at 4 years post-operative, although this cementation was used in only 24 of 964 cases [5]. Schuroff et al. conducted a radiographic evaluation of cementation using a posterolateral surgical approach [22]. Compared to their results we identified a higher percentage of Barracks cementation type A and B in our series. Our analysis demonstrated higher lucency (%) in Gruens Zone 1, 13, 14, and lower lucency (%) in zones 2–12. Burston et al. reported 15-year follow-up results using the posterolateral/transgluteal approach [23]. In their report, 72% of cases qualified to Barrack type A, which was much higher compared to our series. However, we did not come across any type D cases which they reported in 5% of cases. Also, a long-term follow-up from Japan reported 203 Barrack A cases in a series of 211 cases [13]. They utilised a Hardinge/Transtrochanteric Charnley/Dall approach to implant cemented Charnley-type stems in their series. In Dorr C femurs, the larger uncemented implant required to provide fixation would result in a proportionally large Young’s modulus mismatch which may lead to stress transfer and resulting in thigh pain [24]. Hence, we preferred to use cemented stems in these cases.

One of the limitations of this report is the retrospective nature of this study. The potential for measurement errors must be considered, as the radiographic analysis was conducted by a single experienced orthopaedic fellow. The Barrack grading has shown limited inter-and intra-observer agreement [25]. As we used uncemented acetabular cups in all our cases, we are unable to comment on the cementation of the acetabular side using this approach. Also, the duration of follow up is short to comment on lucency lines around the stem. Direct anterior hip replacements are known to have a steep learning curve with complication rates falling with increasing surgeon experience [26]. All operations in our series were performed by a DAA fellowship-trained surgeon, which may have positively influenced postoperative outcomes. Implant choice was based on intraoperative bone quality, thus introducing a potential selection bias. As the surgeon has consistently used one implant system throughout this series, learning curves were minimal, thus minimizing possible complications.

## Conclusion

Our results suggest that cementation in DAA may be performed satisfactorily using appropriate surgical techniques utilizing the bikini incision on a standard operating table. Future prospective comparative studies are recommended to corroborate these findings.

## Conflicts of interest

The authors declare that they have no conflicts of interest in relation to this article.
